# Scope and inclination of voluntary service for urban community‐living older adults provided by volunteers with nursing background: A qualitative study

**DOI:** 10.1111/hex.13990

**Published:** 2024-02-17

**Authors:** Lei Huang, Hongyan Wu, Fengjian Zhang, Xiao Peng, Lin Guo, Lulu Liao, Mengyun Hu, Shuang Wang, Chunyan Guan, Yilan Liu

**Affiliations:** ^1^ Department of Nursing, Union Hospital,Tongji Medical College Huazhong University of Science and Technology Wuhan China; ^2^ School of Nursing,Tongji Medical College, Huazhong University of Science and Technology Wuhan China; ^3^ Health Science Center, Yangtze University Jingzhou China; ^4^ School of Sociology Huazhong University of Science and Technology Wuhan China

**Keywords:** community‐living, nursing, older adult, qualitative study, voluntary service

## Abstract

**Background:**

With the growing challenge of an aging population, addressing the needs of elderly individuals who face living difficulties and lack family support becomes increasingly difficult. Volunteer services are crucial in this context, yet their effectiveness is hindered by unclear service scopes and uncertain volunteer inclinations.

**Aim:**

To explore the role and specific preference of volunteers with nursing backgrounds in support of older adults living in the urban community.

**Design, Setting and Participants:**

A descriptive qualitative study was conducted between September and October 2022. Twenty‐three participants (hospital nurses [10], community nurses [4], nursing teachers [4] and nursing students [5]) were selected. Data analysis followed conventional content analysis.

**Results:**

Nine major themes were identified based on interview data. Four themes described the service scope of nursing volunteers: (1) environment domain, (2) physiological domain, (3) psychosocial domain and (4) health‐related behaviours domain. Another five themes highlighted the service inclination of these volunteers: (1) service frequency, (2) service duration per person/time, (3) service coverage, (4) service place and (5) service object.

**Conclusion:**

This study clarifies the service focus and scope of necessary support for volunteers, exploring the potential service capabilities of scarce volunteers to the greatest extent. Meanwhile, the results of this study also provide a foundation for stakeholders to fully exploit the synergy. The important findings of this study will help the government and relevant authorities better understand the service attributes of nursing volunteers, allowing them to develop detailed training plans and provide nursing volunteers with targeted support and assistance to meet the health expectations of urban community‐living older adults in need.

**Patient or Public Contribution:**

Developing research questions, participation and conduct and provision and interpretation of evidence.

## 1 INTRODUCTION

The aging of the world's population is gradually accelerating. The number of people over 60 worldwide is expected to double by 2050.^1^ Due to the imbalance of population development policies and the large population base, China's aging problem is more serious.^2^ According to data released by the National Bureau of Statistics in 2020, the number of older adults over 60 in China reached 264 million, accounting for 12.6% of the total population.^3^ Unfortunately, as a developing country with an inadequate social security system, the aging population has added to the social burden of caring for the elderly. Regular long‐term care facilities are scarce, and most older adults rely on family care.^4^

In recent years, advancements in China's social economy have unveiled a range of increasingly complex needs among the elderly. This diversity not only encompasses basic care requirements but extends to more sophisticated demands for psychological and medical support.^5^ However, as young people have to devote more energy to work and childcare,^6^ many elderly people lack family support. A previous study showed that about 55% of the elderly in China reported their unmet care needs.^7^ This is especially true for elderly individuals with special difficulties, such as chronic diseases or living alone, who require additional social care and assistance. In this case, the advantages of voluntary service are becoming increasingly prominent as an effective supplement to the social pension system.

### 1.1 Background

Voluntary service is defined as free public service done to society or others by volunteers, voluntary service organizations or other organizations.^8^ Volunteers can offer services in various ways, including spontaneous, intermittent, organized and long‐term, but they must adhere to the principle of being free and beneficial.^9^ Since gaining prominence in the 21st century, volunteer services in China have become increasingly vital in providing social assistance to the elderly, demonstrating a significant surge in activity and impact.^10^ There is some evidence that voluntary activities are closely related to the health of the elderly.^11^ However, unlike in Western countries, the scale of volunteers' participation in social care provision for the elderly in China is far from enough.^12^ To this end, the ‘2030 Plan Outline for a Healthy China’ emphasized the policy guidance of improving the social participation mechanism and developing volunteer services for the elderly to encourage more people to participate.^13^

Research conducted in China, as well as internationally, indicates that limited public engagement in volunteer services often stems from insufficient policy guidance and low participant motivation.^14^, ^15^ Additionally, the lack of platform support and strategic direction for elderly voluntary services contributes to these challenges.^16^, ^17^ As a result, volunteers frequently encounter difficulties, grappling with how to effectively start and make a meaningful impact. Moreover, addressing the needs of the elderly, which frequently encompass psychological and medical aspects, adds another layer of complexity to volunteer efforts.^5^

Since nursing workers have more interdisciplinary knowledge reserves and service experiences,^18^ encouraging volunteers with nursing backgrounds to participate in assistance services for the elderly will be more helpful in improving their quality of life.^19^ Meanwhile, if nursing workers participate in voluntary organizations, other members also can obtain professional support and guidance to benefit more older adults. However, no published research exists on the content framework or preferences for volunteers with nursing backgrounds to provide services to elderly people in the community. Previous studies involving the elderly living in communities mainly focused on the driving force or resistance to engaging in volunteer service, as well as the effect of volunteers.^3^, ^20^, ^21^ In addition, in China, urban home‐based older adults often require more social assistance than rural older adults because they mostly live in high‐rise buildings rather than bungalows, which can lead to social isolation and difficulty in obtaining support and assistance from neighbours and other social resources.^22^ Overall, the results of this study have insightful implications for volunteers with a nursing background to do a good job in the preparatory work of serving the elderly living in urban communities, as well as providing a foundation for stakeholders to understand the service orientation of these volunteers to play a good role in synergy.

### 1.2 Aim

This study aimed to explore the role and preference of volunteer services with nursing backgrounds for urban community‐living older adults.

## 2 METHODS

### 2.1 Design

A descriptive qualitative design was conducted because little information is known about the scope of voluntary service provided by volunteers with nursing backgrounds.^23^ Here, the method of data collection and analysis is used to thoroughly present the subjective nature of respondents in simple language, increasing the agreement among researchers and participants.^24^

### 2.2 Participants

The study was conducted in Wuhan, Hubei Province, from 13 September to 23 October 2022. Participants were selected using purposive sampling methods, including criterion and maximum variation sampling. Contact information for heads of various volunteer service organizations or projects was obtained from the official website of volunteer service in China (http://chinavolunteer.mca.gov.cn/site/home). Recruitment for this study was carried out through invitations and recommendations from the individuals in charge. Additionally, recruited participants were introduced to other eligible participants, following a snowball sampling method. The following criteria were used for inclusion: (i) individuals with a nursing professional background; (ii) individuals aged 18 years and above and (iii) having at least one experience of providing volunteer service for urban community‐living older adults. Participants were excluded if they: (i) were not interested in our interview topic and (ii) had experience volunteering for older adults in rural areas or nursing homes.

### 2.3 Data collection

Data were collected through face‐to‐face or telephone interviews by the first author, a doctoral student in the nursing field. The face‐to‐face interview was arranged in a quiet room in the participant's work area, such as an idle office, ensuring the quality of the interview and the privacy of the interviewees. The option of telephone interview was provided to encourage participation from volunteers with nursing backgrounds who could not meet due to the COVID‐19 epidemic or busy work. Video phones and WeChat‐based video calls were employed to ensure that an equal amount of information was collected as in face‐to‐face interviews. The entire interview process was recorded using a recording pen to enable accurate transcription. During the study, the first author and another researcher were involved in the interview process; extensive notes were made by the interviewer to capture perspectives as it occurred,^25^ and the interviews were also observed and audio‐recorded by another researcher. During each interview, only the first author, the participant and another researcher were present. If the information obtained in the interview was difficult to identify or understand, the researcher would give timely feedback to the interviewee for clarification. An interview guide including six open‐ended qualitative questions was used to collect interview data (see Table 1). Socio‐demographic data such as gender, age, research field, and service life were also collected (see Table 2). All interviews were recorded using a recording pen and then transcribed into Chinese using IFLYTEK hearing transcription software. Two bilingual translators translated the collated data into English to ensure that the meaning remained unchanged. The interviews lasted between 20 and 60 min.

**Table 1:** Interview questions.

1. What kind of voluntary service have you provided for older adults?
2. What other support or help do you think you can provide for older adults within your ability?
3. How often do you think it is acceptable to help older adults? How long is it appropriate for each person at a time?
4. Where do you think it is most feasible to help older adults? How much time is it acceptable to spend on the road?
5. What characteristics of older adults do you think need more help from volunteers?
6. Do you want to add something? Are there any critical aspects we did not talk about so far?

**Table 2:** Participant characteristics (*n* = 23).

Characteristics	Number (%)
Gender (female), *n* (%)	21 (91.3)
Age (years) (mean ± SD)	37.87 ± 13.11
Occupation	
Student	5 (21.7)
Teacher	4 (17.4)
Nurse	14 (60.9)
Affiliated institutions	
University	9 (39.2)
Community hospital	4 (17.4)
Large‐sized hospital	5 (21.7)
Medium‐sized hospital	2 (8.7)
Small‐sized hospital	3 (13.0)
Education	
College degree or under	5 (21.7)
Bachelor	12 (52.2)
Postgraduate or above	6 (26.1)
Position	
Director	2 (8.7)
Head nurse	5 (21.7)
Others	16 (69.6)
Research field	
TCM nursing	3 (13.0)
Geriatric nursing	4 (17.4)
Clinical nursing	4 (17.4)
Others	12 (52.2)
Work seniority (years)	
0–1	5 (21.7)
2–10	4 (17.4)
>10	14 (60.9)
Service life	
0–2	5 (21.7)
3–5	12 (52.2)
>5	6 (26.1)

Abbreviation: TCM, traditional Chinese medicine.

### 2.4 Data analysis

Before data analysis and coding, the first author immersed himself in the data, listening to the recordings multiple times. The text data transcribed by IFLYTEK hearing transcription software was then compared and verified with the audio data several times to correct the transcription error. The first author and another researcher independently extracted and classified the meaningful coded content in the transcript using the conventional content analysis method, forming related themes and subthemes.^26^ In traditional content analysis, encoding categories are directly derived from textual data, avoiding the use of preconceived categories and allowing categories and names to emerge naturally from the data.^27^ After regular online conferences, research group members discussed and compared the differences in the encoding contained in the newly identified subthemes from the two researchers to reach a consensus. Themes and codes were constantly revised to ensure alignment with the data and research objectives. During data collection, when the interview data of volunteers from the same occupation approached saturation, 2–3 additional volunteers were interviewed to ensure that the data reached saturation and no new subthemes emerged.^28^ The interview ended after confirming that no further information was obtained. In classifying the coding rigorously, themes and subthemes were categorized based on the problem classification scheme of the Omaha system.^29^ Summaries of themes and subthemes are presented in Tables 3 and 4.

**Table 3:** Summary of themes and subthemes (scope of voluntary service).

Themes	Subthemes	Codes
Environment domain	Household hygiene	Clean rooms and tidy up
	Furnishings maintenance	Maintenance of household supplies
	Environmental safety	Measures to avoid falls
Physiological domain	Life assistance	Substitute or accompany the procurement
	Life guidance	Lifestyle adjustment
		Use of intelligent devices
	Information support	Correctly understand nursing homes
		Answers to puzzles in life
Psychosocial domain	Chat	Chat around some topics
	Psychological guidance	Psychological assessment and guidance
		Online follow‐up support
	Cognitive restructuring	Change their negative view of disease or death
		Discern between true and false
	Family maintenance	How to get on family members
		Remind children to care about their parents
	Entertainment organization	Add interest to life
	Interaction at home	Festival or birthday interaction at home
	Interest cultivation	Encourage them to amuse themselves
	Intercourse encouragement	Go out to socialize
Health‐related behaviours domain	Risk assessment	Risk assessment of life safety
	First‐aid popularization	Publicity of first‐aid knowledge
	Health Education	Health care or disease prevention education
	Health service	Disease surveillance
		Health care
		Wound or tube care
	Targeted guidance	Individual health guidance
	Medication management	Guide the use and management of drugs
	Accompany for medical treatment	Accompany physical examination or see a doctor

**Table 4:** Summary of themes and subthemes (Inclination of voluntary service).

Themes	Subthemes	Codes
Service frequency	Interval within one month	Once a week
		At least once a month
	Near a quarterly interval	Once every 2 or 3 months
Service duration per person/time	A hasty stay	Within 1 h
	A well‐planned stay	Between 2 and 3 h
		Half a day's duration
Service coverage	Short distance	Arrive within half an hour
		Arrive within 1 h
	Moderate distance	Arrive in less than 2 h
Service place	Offline	At home
		In the community
	Online	Call or WeChat
Focused groups	Poor health	Disease ridden
		Mobility difficulties
	Living alone	Living alone
	Social Disorders	Uncommunicative
		Only stay at home

### 2.5 Trustworthiness of the study

All researchers involved in this study have undergone comprehensive training in qualitative research methodologies and have extensive practical experience. To prevent potential bias, interviewers had no prior relationships with the participants. The point of data saturation was meticulously determined by two independent researchers who evaluated and compared the emerging subthemes. This process ensured that no new relevant data was appearing, indicating a thorough exploration of the topic. To guarantee the reliability of the data collected, we engaged in participant validation by returning the transcribed text to the participants for confirmation. Our researchers deeply immersed themselves in the data, conducting continuous and meticulous analysis and comparison. Following the principle of maximum variation sampling, the study included diverse participant characteristics, such as different occupational groups and wide age ranges, to seek data transferability.

## 3 RESULTS

### 3.1 Sample characteristics

This study included 23 volunteers, which included 10 nurses from various hospitals, 4 community nurses, 4 nursing teachers and 5 nursing students. All participants had direct experience providing voluntary services to older adults living in urban communities. The average age of the participants was 37.9 years (range: 20–60 years), and most were female (*n* = 21, 91%). Most participants had more than 3 years of volunteer service experience (*n* = 18, 78%) (see Table 2 for a summary of demographics; detailed information is provided in Supporting Information S1: Appendix I).

### 3.2 Identified themes

The participants were numbered P1–23 in this study. The themes and codes derived from the interviews are shown in Tables 3 and 4. Supplementary tables with additional examples of related sentences/phrases are presented in Supporting Information S1: Appendices II and III.

### 3.3 Category 1: Scope of voluntary service

#### 3.3.1 Themes 1: Environment domain

While participants in this study excel in providing medical‐related services, some also emphasized the importance of improving the living environment for the elderly. This includes tasks such as cleaning and organizing rooms, assisting with household chores, and implementing fall prevention measures. These efforts enhance the elderly's comfort and safety.

##### Household hygiene

Sometimes, if there is no suitable penetration point for home service, some participants may clean the room. After all, this is very practical. ‘I contacted the community, cleaned the older adult's house together, and designed a simple room layout’ (P18).

##### Furnishings maintenance

However, during the family visit, a few participants found that some household items in older adults' homes were damaged, and they offered to help send for repairs or seek assistance from the community. ‘Some things may be broken, such as the TV, gas range or home switches. We will try to repair them or give feedback to the community’ (P6).

##### Environmental safety

Nursing work helps cultivate acute insight. Based on this professional quality and clinical experience, some participants identified potential fall risks in time in older adults' homes and put forward feasible suggestions. ‘Some older adults also need to be reminded of hidden dangers in the bathroom, such as slippery ground, doorsill or no handrails in the toilet’ (P19).

#### 3.3.2 Themes 2: Physiological domain

Providing essential items, life guidance, and information support to elderly individuals facing living difficulties greatly enhances their sense of happiness.

##### Life assistance

Some participants offer such basic services. ‘What I used to do most was to buy vegetables for the elderly. Run errands with others to get medicine’ (P17).

##### Life guidance

Many participants mentioned life guidance, including leading a healthy lifestyle and teaching the use of intelligent devices. ‘I will teach them some smart devices, such as mobile phones, if they do not know how to use them’ (P5).

##### Information support

A few participants also mentioned their support in life information consultation, which often involves older adults' concerns in life. ‘Many elderly people do not sleep well, and they may have sleep disorders such as OSAS. I will answer any questions they may have’ (P7).

#### 3.3.3 Themes 3: Psychosocial domain

In addition to life, the spiritual world of the elderly is also highly valued by most participants. Volunteers can enhance the psychological well‐being and social adaptability of elderly individuals through activities like companionship, family relationship support, and adding enjoyment to their lives.

##### Chat

Many participants had emotional exchanges with the elderly about breaking news or interesting topic. ‘I think volunteers can bring them something new and let them feel the real world’ (P8).

##### Psychological guidance

To improve the psychological problems of the elderly, many participants emphasized the importance of identifying psychological abnormalities and providing targeted psychological counselling and online follow‐up support for the elderly. ‘We can fully utilize our professional advantages, understand their family situation, detect psychological issues, such as anxiety and depression, and provide appropriate psychological counselling’ (P14).

##### Cognitive restructuring

Many participants described hidden findings that must be addressed when providing voluntary services. Even older adults without cognitive impairment often find it challenging to distinguish between pseudoscience or false advertising. This difficulty can lead not only to misconceptions in their cognitive or behavioural patterns but also to economic losses. Similarly, when faced with negative events or uncontrollable sources of stress, they may struggle to approach the problem with the right mindset. ‘As for the false or unscientific knowledge acquired by the elderly through some media, I will help them identify the truth’ (P6).

##### Family maintenance

It is common for the elderly to interact infrequently with their families, especially with their children. Some participants believed that volunteers needed to play a role in maintaining bilateral family relations. ‘Some elderly people are unhappy throughout the day because they do not get along with their children or partners. We will also assist them’ (P22).

##### Entertainment organization

Social isolation among the elderly is extremely common, particularly in urban areas. The organizational activities have high requirements for the preparation work, which are difficult to meet but can be oriented to a broader range of elderly groups. Student volunteers mentioned this type of service. ‘We will bring them some chorus performance of revolutionary songs’ (P1).

##### Interaction at home

It is considered that the elderly are more likely to feel lonely and depressed on important festivals or anniversaries. Some participants hold warm celebrations at home for some special older adults, such as those who live alone. ‘We accompany the elderly to make moon cakes and dumplings at the Mid Autumn Festival and dumplings together at the Lantern Festival’ (P12).

##### Interest cultivation

Many participants encouraged the elderly to enjoy themselves. ‘If they are educated, we will encourage them to turn on the TV or listen to the radio, and sometimes teach them to play with their smartphones’ (P19).

##### Intercourse encouragement

Some participants also emphasized the importance of socializing with the elderly, which is very beneficial for them to maintain a good mentality and build confidence in life. ‘The elderly also need to have their social circle. Usually, they should be urged to go downstairs to communicate with their neighbours or dance square dances together’ (P15).

#### 3.3.4 Themes 4: Health‐related behaviours domain

As volunteers with a nursing background, maintaining or promoting health‐related behaviours of the elderly is the key link of voluntary services. All participants emphasized health promotion or disease management measures, such as first‐aid popularization, health service, and medication management.

##### Risk assessment

Some participants believed that timely risk assessment was equally important compared to specific services. ‘We can make some assessments for the elderly, such as fall and pressure ulcer risk’ (P16).

##### First‐aid popularization

Some participants highlighted that the vulnerable group of the elderly should master some easy‐to‐operate first‐aid technologies. ‘What we carry out most is the first aid propaganda for the elderly in the community, such as CPR, Heimlich, bandaging, and transportation’ (P7).

##### Health education

Many participants believed centralized health education in communities would benefit older adults more. ‘Health education primarily focuses on chronic disease management, winter cold prevention, vaccination urging, prevention of pressure sores and falls, and some lifestyle or exercise precautions’ (P12).

##### Health service

Furthermore, many participants tended to provide healthcare services that depended on their professional skills. For example, ‘I often go to help them measure BP and GLU’ (P9).

##### Targeted guidance

Many participants also provided targeted guidance and advice on diseases of the elderly. ‘Based on our specialty, we will give some older adults with chronic diseases guidance on preventing complications, such as sputum excretion from a lung infection, and how to prevent thrombosis in hemiplegic limbs’ (P20).

##### Medication management

In addition to the regular medication guidance, a few participants provided more elaborate services, such as sorting and classifying drugs and recommending low‐cost alternative medicines. ‘If I find that some medicines in the homes of elderly people have expired, I will assist them in sorting the medications, labeling them, and instructing them on when to use them’ (P12).

##### Accompany for medical treatment

A few participants also shared their experiences of accompanying the elderly to go out for physical examination or medical treatment. ‘When some patients’ families are not around, sometimes I will accompany them to the physical examination or registered medical treatment’ (P13).

### 3.4 Category 2: The inclination of voluntary service

#### 3.4.1 Themes 5: Service frequency

Volunteers seem to be inclined to provide more frequent volunteer services.

##### Interval within 1 month

Most participants believed they could go at least once a month, and nearly half could go at least once a week. ‘I think once a month is not a problem’ (P2).

##### Near a quarterly interval

Very few participants felt they could volunteer at least once every 2 months. ‘I think it is not a problem for me to go once every two or three months’ (P11).

#### 3.4.2 Themes 6: Service duration per person/time

More volunteers tend to spend an appropriate amount of time on individuals.

##### A hasty stay

Most participants believed they would not spend more than 1 h on each person at a time but not less than half an hour. ‘I believe it is reasonable to allow between 30 min and an hour’ (P4).

##### A well‐planned stay

A few participants can provide services of 2–3 h or more than half a day, each person at a time. ‘I prefer two to three hours’ (P17).

#### 3.4.3 Themes 7: Service coverage

Most volunteers are unwilling to spend too much unnecessary time to reach the destination.

##### Short distance

Most participants believed it was acceptable to arrive within 1 h, and more than half of them preferred to arrive within half an hour. ‘It is better to take the bus within half an hour’ (P15).

##### Moderate distance

Individual participants are the only ones willing to spend more time on the road. ‘It takes less than 2 h to get there using public transportation’ (P5).

#### 3.4.4 Themes 8: Service place

Although online service is also a popular form of service, more volunteers prefer family‐based offline service. Online services, in the context of our study, refer to various digital platforms and internet‐based solutions that facilitate the provision of healthcare, social interaction, and other forms of support to the elderly.

##### Offline

Most participants believed it is better to provide volunteer services offline, and more than half of them preferred to serve the elderly at home rather than in the community. ‘Although online is more convenient, offline provides a more intimate experience for the elderly, which is more practical’ (P15).

##### Online

However, many participants still encourage the development of online services or as an effective complementary form of offline services. ‘I prefer online because evidence and a record can be left to avoid disputes’ (P21).

#### 3.4.5 Themes 9: Focused groups

Although the elderly who are not good at speaking or going out for communication also need attention, volunteers are more inclined to serve the elderly who live alone or have difficulty in action.

##### Poor health

Most participants believed that the elderly who lived alone or had poor health should be prioritized for assistance. ‘The elderly with chronic diseases or long‐term bedridden need assistance most’ (P4).

##### Living alone

Likewise, most participants believed that the elderly who lived alone should also be a key focus of attention. ‘I believe it is most necessary for empty nesters’ (P23).

##### Social disorders

Besides, very few participants believed that the elderly who could not go out at home or were not good at communication needed more attention. ‘The elderly who do not talk outside need more attention’ (P8).

## 4 DISCUSSION

This is the first study to examine the scope and inclination of voluntary service for urban community‐living older adults provided by volunteers with a nursing background. These service items addressed nearly every aspect of the elderly's life, including environmental, physiological, psychosocial and health‐related behaviours.

All of our participants indicated health‐related service habits that benefited from their professional advantages. Chronic diseases are often quite difficult for the elderly. However, volunteers without professional training are often dependent on assistance from others. Therefore, the findings of this study will guide not only various practitioners in the field of nursing to actively engage in elderly volunteer activities but also benefit other social volunteers. Unlike other service behaviours, risk assessment mentioned by some participants tends to be a guidance method. Based on the risk assessment of underlying chronic diseases, pressure sores or falls, it is more conducive to implementing the targeted intervention. Similarly, several previous studies confirmed the feasibility of using volunteers to screen the health risks of the elderly.^30^, ^31^ However, some other participants also mentioned targeted guidance aimed at improving the obvious symptoms of the elderly, such as providing advice on preventing complications of chronic diseases. Our findings also revealed that, while encouraging volunteers to participate in emergency rescue appears to be more effective,^32^ it is also a good choice to popularize first‐aid knowledge and demonstrate operations to the elderly, given their interest in acquiring such knowledge and limited support resources available. Interestingly, A small group of participants suggested sorting out and labelling the drugs stored in disorder for the elderly to avoid their incorrect medication. Meanwhile, considering the generally poor economic situation of the elderly, volunteers can recommend low‐cost alternative medicines to them when necessary. Although the services mentioned above fall within the professional scope of nursing staff, they provide new ideas for volunteering for home‐based older adults. Therefore, volunteers should be encouraged to provide services based on their professional strengths, such as drug management and health monitoring.

Given the actual needs of the elderly, most participants may be more inclined to provide health education or health services for the elderly. Depending on the circumstances, volunteers can choose centralized education or one‐on‐one guidance, such as conducting lectures in community activity rooms. Some professional health promotion services, such as relaxing massage and trauma management, are advocated, but volunteers also need to do some preparation work. Furthermore, a physical examination or medical treatment accompanied by volunteers is beneficial. According to a recent qualitative study, many older persons want to receive substantive medical services such as illness guidance, health monitoring, and health treatment without leaving their homes.^5^ In this context, the present findings strongly support the health‐related needs of the community‐living elderly. Besides, volunteer training should encourage volunteers to acquire more extensive service practices as well as strive to spark the interest of older persons.

Another important theme was found to be volunteer service involving the psychosocial domain. Although previous studies demonstrated the necessity of psychosocial intervention, few specific behavioural guidances based on empirical experience were proposed.^33^ The findings of this study will provide evidence for volunteers to serve in the field of psychosocial services. To capture and address the psychological problems of the elderly, many participants mentioned aimless chatting, which can be to discuss interesting topics or share news. If psychological abnormalities are identified, volunteers should be encouraged to promptly carry out psychological guidance based on assessment and keep online follow‐up if necessary. A few participants also noticed that some older adults might have negative attitudes and coping styles toward death or disease. According to an Iranian study, irrational beliefs and attitudes toward death would directly affect the quality of life of the elderly.^34^ Conversely, encouraging a positive aging attitude may help older people improve their emotional health.^35^ Therefore, helping the elderly rebuild their cognition should also be a focus of psychological intervention of volunteers, which should also include some wrong understandings and concepts in their lives. Furthermore, some participants also realized that the elderly who are estranged from society are more vulnerable to the psychological impact of bad family relations and provided solutions. In summary, providing mental health training for volunteers is essential to better identify and address the psychological issues of the elderly while also promoting a positive attitude toward aging and helping the elderly establish a positive outlook on life.

A unique finding was that some participants ‘upgraded’ their services to please the elderly, such as painstakingly preparing entertainment programmes and celebrating birthdays or holidays for the elderly. Unfortunately, such intimate interactions are rarely reported. In enriching the life of the elderly, some participants encourage them to entertain themselves, such as by watching TV and surfing the Internet. Facts have proved that Internet surfing is conducive to increasing social contact and improving mental health.^36^ Moreover, physically fit elderly should be advised to go out and socialize more. A recent study from the United States also suggested that student volunteers establish relationships with the elderly through a longitudinal telephone plan to reduce their social isolation, especially during the COVID‐19 pandemic.^37^ Therefore, in addition to encouraging elderly people to enrich their spiritual life, volunteers can also take some interactive activities when necessary to enhance the happiness experience of the elderly.

Although the participants in this study were more inclined to provide high‐level support related to physical and mental health, many participants also observed some potential difficulties and hidden dangers in the daily lives of the elderly that must be addressed immediately. Regarding physiology, the service scope mentioned by participants is generally consistent with the assistance needs of urban home‐based elderly.^5^ Given the extreme views of many elderly people on nursing homes, it is necessary to help them develop an objective understanding of nursing homes in due course. In short, it is necessary for volunteers to first consult relevant materials and then provide accurate information support for the elderly to solve their confusion.

Similarly, in terms of the living environment, participants often provide services that meet the expectations of urban home‐based elderly.^5^ However, due to their professional sensitivity, some participants emphasized the potential risk factors that may lead to falls in the living environment, such as slippery ground, doorsill or no handrails in the toilet. After all, falls are common among the elderly over the age of 65, and the harm they cause is obvious, so they deserve special attention.^38^

This study also paid attention to the service inclination of assisting community‐living elderly among nursing volunteers for the first time. Surprisingly, most participants expected to provide frequent door‐to‐door services, such as once a week or a month. After all, the high‐pressure environment in the nursing industry makes it difficult for them to spare time after their usual work.^39^ Although our participants preferred to serve the elderly in person, they also emphasized the need to spend as little time on the road as possible. However, it is worth noting that online services appear to be more feasible, particularly during the COVID‐19 epidemic.^37^ Furthermore, given the serious imbalance between the supply and demand of voluntary services, our participants suggested that priority should be given to the elderly who live alone, have difficulty in activities, and are poor communicators. Finally, based on the important findings of this study, healthcare authorities and managers can also better understand the preferences of nursing practitioners in volunteering to provide a reference for establishing a volunteer service team under the guidance of the scientific service framework while fully promoting the collaborative role of other organizations or departments.

This study has several limitations. First, the research results are generated in a small sample of participants from several institutions in a metropolis, thus limiting the promotion of our research results in other environments. Second, we could not include more men in our study due to the small number of men in nursing. Moreover, all data were collected during the COVID‐19 pandemic, affecting the transferability of research results after the end of the pandemic.

## 5 CONCLUSION

This study collects information from the perspective of volunteers with nursing backgrounds to enhance their service capabilities and efficiency, addressing the critical needs of elderly community residents. Clearly, unlike the service nature of homecare or continuing care, this study is intended to address the critical needs of elderly community residents who urgently need help in all aspects of life. The crucial finding in this study is both similarities and differences between the service orientation of nursing volunteers and the demands of the elderly will help to develop scientific volunteer training strategies.

In short, this study clarifies the service focus and scope of necessary support for volunteers, exploring the potential service capabilities of scarce volunteers to the greatest extent. Meanwhile, the results of this study also provide a foundation for stakeholders to fully exploit the synergy. The important findings of this study will help the government and relevant authorities better understand the service attributes of nursing volunteers, allowing them to develop detailed training plans and provide nursing volunteers with targeted support and assistance to meet the health expectations of urban community‐living older adults in need.

## AUTHOR CONTRIBUTIONS

**Lei Huang**: Conceptualization; methodology; software; data curation; supervision; resources; project administration; formal analysis; validation; visualization; writing—original draft; investigation. **Hongyan Wu**: Conceptualization; writing—review and editing. **Fengjian Zhang**: Methodology. **Xiao Peng**: Methodology. **Lin Guo**: Methodology. **Lulu Liao**: Methodology. **Mengyun Hu**: Data curation. **Shuang Wang**: Data curation. **Chunyan Guan**: Writing—review and editing. **Yilan Liu**: Writing—review and editing.

## CONFLICT OF INTEREST STATEMENT

The authors declare no conflict of interest.

## ETHICS STATEMENT

The research protocol was approved by the Medical Ethics Committee of Tongji Medical College, Huazhong University of Science and Technology (Ethical review No. S053). Before the interview, all participants were informed of the purpose of the study and the necessity of recording and were asked for verbal or written consent.

## Supporting information

Supporting information.

## Data Availability

The data that support the findings of this study are available in the supplementary material of this article.
