# Supplementary material for: Scope and inclination of voluntary service for urban community‐living older adults provided by volunteers with nursing background: A qualitative study
Source: Health Expect. 2024 Feb 17;27(1):e13990. doi: 10.1111/hex.13990 (PMC10874248; doi:10.1111/hex.13990)
Supplement: Supplementary file 1 — Supporting information. [file HEX-27-e13990-s001.docx]

**Appendix I.**

Detailed Participant characteristics (n=23)

| Interview method | Participant no. | Age | Gender | Occupation | Affiliated institutions | Education | Position | Work seniority | Research field | Service life |
| --- | --- | --- | --- | --- | --- | --- | --- | --- | --- | --- |
| Telephone | 1 | 21 | Female | Student | University | MBBSc | / | / | TCM Nursing | 2 years |
| Telephone | 2 | 21 | Female | Student | University | MBBSc | / | / | TCM Nursing | 3 years |
| Telephone | 3 | 20 | Female | Student | University | MBBSc | / | / | Geriatric Nursing | 3 years |
| Face-to-face | 4 | 29 | Male | Student | University | MD | / | / | Nursing management | 5 years |
| Face-to-face | 5 | 20 | Female | Student | University | JCc | / | / | Clinical nursing | 3 years |
| Telephone | 6 | 30 | Female | Teacher | University | MDc | / | 2 years | Community nursing | 5 years |
| Face-to-face | 7 | 59 | Female | Teacher | University | MSN | Director | 41 years | Clinical nursing | 6 years |
| Face-to-face | 8 | 39 | Female | Teacher | University | MSN | Director | 14 years | Nursing management | 6 years |
| Face-to-face | 9 | 32 | Male | Teacher | University | MSN | / | 6 years | Chronic disease care | 5 years |
| Face-to-face | 10 | 35 | Female | Nurse | Community Hospital | MBBS | / | 14 years | Community nursing | 2 years |
| Face-to-face | 11 | 50 | Female | Nurse | Community Hospital | JC | / | 30 years | Clinical nursing | 5 years |
| Face-to-face | 12 | 22 | Female | Nurse | Community Hospital | MBBS | Group leader | 9 years | Geriatric Nursing | 5 years |
| Telephone | 13 | 39 | Female | Nurse | Community Hospital | MBBS | / | 19 years | Psychological care | 3 years |
| Telephone | 14 | 51 | Female | Nurse | Large-sized hospital | MBBS | / | 30 years | Surgical Nursing | ＞10 years |
| Telephone | 15 | 33 | Female | Nurse | Large-sized hospital | MBBS | / | 12 years | Wound care | 3 years |
| Telephone | 16 | 42 | Female | Nurse | Large-sized hospital | MBBS | Head nurse | 23 years | Cancer Nursing | 1 years |
| Telephone | 17 | 58 | Female | Nurse | Large-sized hospital | JC | / | 38 years | Surgical Nursing | 6 years |
| Telephone | 18 | 29 | Female | Nurse | Large-sized hospital | MSN | Officer | 5 years | Psychological care | 1 years |
| Telephone | 19 | 40 | Female | Nurse | Medium-sized hospital | MBBS | Head nurse | 20 years | Geriatric Nursing | 6 years |
| Telephone | 20 | 39 | Female | Nurse | Medium-sized hospital | MBBS | Head nurse | 15 years | Surgical Nursing | 3 years |
| Telephone | 21 | 50 | Female | Nurse | Small-sized hospital | JC | Head nurse | 31 years | Geriatric Nursing | 4 years |
| Telephone | 22 | 52 | Female | Nurse | Small-sized hospital | MBBS | Head nurse | 34 years | TCM Nursing | ＞10 years |
| Telephone | 23 | 60 | Female | Nurse | Small-sized hospital | JC | / | 10 years | Clinical nursing | 2 years |

**Appendix Ⅱ.**

Summary of themes and sub-themes (Scope of voluntary service)

| Themes | Sub-themes | Codes | Example of related sentences/phrases |
| --- | --- | --- | --- |
| Environment domain | Household hygiene | Clean rooms and tidy up | ‘I contacted the community, cleaned the older adult's house together, and designed a simple room layout’ (P18) |
|  | Furnishings maintenance | Maintenance of household supplies | ‘Some things may be broken, such as the TV, gas range or home switches. We will try to repair them or give feedback to the community’ (P6) |
|  | Environmental safety | Measures to avoid falls | ‘Some older adults do not have a lamp at their bedside, and I suggested a small table lamp. And a small stool should be placed in the bathroom’ (P12) |
|  |  |  | ‘Some older adults also need to be reminded of hidden dangers in the bathroom, such as slippery ground, doorsill or no handrails in the toilet’ (P19) |
| Physiological domain | Life assistance | Substitute or accompany the procurement | ‘What I used to do most was to buy vegetables for the elderly. Run errands with others to get medicine’ (P17) |
|  | Life guidance | Lifestyle adjustment | ‘I will correct their bad habits and give them some guidance, such as diet, drinking water, exercise, and sleep’ (P8) |
|  |  | Use of intelligent devices | ‘I will teach them some smart devices, such as mobile phones, if they do not know how to use them’ (P5) |
|  | Information support | Correctly understand nursing homes | ‘Many elderly people believe that they will be sent to a nursing home only when no one cares. We should share some positive cases to help them establish a correct understanding’ (P19) |
|  |  | Answers to puzzles in life | ‘Many elderly people do not sleep well, and they may have sleep disorders such as Obstructive Sleep Apnea. I will answer any questions they may have’ (P7) |
| Psychosocial domain | Chat | Chat around some topics | ‘Some older adults are not good at communicating with their children but have a strong sense of loneliness. They may need someone to talk to’ (P17) |
|  |  | Share news | ‘I think volunteers can bring them something new and let them feel the real world’ (P8) |
|  | Psychological guidance | Psychological counseling based on evaluation | ‘We can fully utilize our professional advantages, understand their family situation, detect psychological issues, such as anxiety and depression, and provide appropriate psychological counseling’ (P14) |
|  |  | Online follow-up guidance | ‘We will occasionally chat with some older adults who have psychological problems by phone or WeChat to improve their mood or mentality’ (P22) |
|  | Cognitive restructuring | Change their negative view of disease or death | ‘Sometimes patients with advanced tumors are very cynical and even discuss death-related negative topics. They must establish a correct and reasonable understanding’ (P17) |
|  |  | Discern between true and false | ‘As for the false or unscientific knowledge acquired by the elderly through some media, I will help them identify the truth’ (P6) |
|  | Family maintenance | How to get on family members | ‘Some elderly people are unhappy throughout the day because they do not get along with their children or partners. We will also assist them’ (P22) |
|  |  | Remind children to care about their parents | ‘If the elderly live alone or employ nursing workers, we will also communicate with their children to pay more attention to them’ (P19) |
|  | Entertainment organization | Add interest to life | ‘We will bring them some chorus performance of revolutionary songs’ (P1) |
|  |  |  | ‘We can play some videos for the elderly who cannot travel, such as sightseeing videos’ (P17) |
|  | Interaction at home | Festival or birthday interaction at home | ‘Sometimes we will go to older adults' homes to accompany them on their birthday’ (P18) |
|  |  |  | ‘We accompany the elderly to make moon cakes and dumplings at the Mid Autumn Festival and dumplings together at the Lantern Festival’ (P12) |
|  | Interest cultivation | Encourage them to amuse themselves | ‘If they are educated, we will encourage them to turn on the TV or listen to the radio, and sometimes teach them to play with their smartphones’ (P19) |
|  | Intercourse encouragement | Go out to socialize | ‘The elderly also need to have their social circle. Usually, they should be urged to go downstairs to communicate with their neighbors or dance square dances together’ (P15) |
| Health-related behaviors domain | Risk assessment | Risk assessment of life safety | ‘We can make some assessments for the elderly, such as fall and pressure ulcer risk’ (P16) |
|  |  |  | ‘I can assess him for common disease risks, such as hypertension, diabetes, and coronary heart disease’ (P21) |
|  | First-aid popularization | Publicity of first-aid knowledge | ‘What we carry out most is the first aid propaganda for the elderly in the community, such as cardiopulmonary resuscitation, Heimlich, bandaging, and transportation’ (P7) |
|  | Health Education | Health care or disease prevention education | ‘Health education primarily focuses on chronic disease management, winter cold prevention, vaccination urging, prevention of pressure sores and falls, and some lifestyle or exercise precautions’ (P12) |
|  | Health service | Disease surveillance | ‘I often go to help them measure blood pressure and Glucose’ (P9) |
|  |  | Health care | ‘Moxibustion, scraping, and some simple acupoint massage’ (P1) |
|  |  | Wound or tube care | ‘Change dressing or maintain pipes for them at home’ (P17) |
|  | Targeted guidance | Individual health guidance | ‘Based on our specialty, we will give some older adults with chronic diseases guidance on preventing complications, such as sputum excretion from a lung infection, and how to prevent thrombosis in hemiplegic limbs’ (P20) |
|  | Medication management | Guide the use and management of drugs | ‘If I find that some medicines in the homes of elderly people have expired, I will assist them in sorting the medications, labeling them, and instructing them on when to use them’ (P12) |
|  |  |  | ‘If I notice any negative symptoms in the elderly, such as anemia, I will advise them to take medication. I'll also give them some low-cost alternative medicines’ (P14) |
|  | Accompany for medical treatment | Accompany physical examination or see a doctor | ‘When some patients' families are not around, sometimes I will accompany them to the physical examination or registered medical treatment’ (P13) |

**Appendix Ⅲ.**

Summary of themes and sub-themes (Inclination of voluntary service)

| Themes | Sub-themes | Codes | Example of related sentences/phrases |
| --- | --- | --- | --- |
| Service frequency | Interval within one month | Once a week | ‘I think once a week is acceptable’ (P4) |
|  |  | At least once a month | ‘I think once a month is not a problem’ (P2) |
|  | Near a quarterly interval | Once every two or three months | ‘I think it is not a problem for me to go once every two or three months’ (P11) |
| Service duration per person/time | A hasty stay | Within 1 hour | ‘I believe it is reasonable to allow between 30 min and an hour’ (P4) |
|  | A well planned stay | Between two and three hours | ‘I prefer two to three hours’ (P17) |
|  |  | Half a day's duration | ‘I think if we go, we will probably stay for half a day’ (P3) |
| Service coverage | Short distance | Arrive within half an hour | ‘It is better to take the bus within half an hour’ (P15) |
|  |  | Arrive within 1 hour | ‘It is acceptable to take the bus for dozens of minutes. You will get irritated if you have to ride the bus for more than an hour’ (P10) |
|  | Moderate distance | Arrive less than 2 hour | ‘It takes less than two hours to get there using public transportation’ (P5) |
| Service place | Offline | At home | ‘Although online is more convenient, offline provides a more intimate experience for the elderly, which is more practical’ (P15) |
|  |  | In the community | ‘It is better to concentrate on volunteering for the elderly in the community because the home is not very safe’ (P5) |
|  | Online | Call or WeChat | ‘I prefer online because evidence and a record can be left to avoid disputes’ (P21) |
| Focused groups | Poor health | Disease ridden | ‘The elderly with chronic diseases or long-term bedridden need assistance most’ (P4) |
|  |  | Mobility difficulties | ‘The elderly who are physically inconvenient to travel need help most’ (P1) |
|  | Living alone | Living alone | ‘I believe it is most necessary for empty nesters’ (P23) |
|  | Social Disorders | Uncommunicative | ‘The elderly who do not talk outside need more attention’ (P8) |
|  |  | Only stay at home | ‘Some older adults often stay in their own homes without going out, which needs attention most’ (P5) |
